# Genetic Diversity and Breeding System of the Pestiferous Subterranean Termite *Reticulitermes flaviceps* Across Shaanxi and Sichuan Provinces

**DOI:** 10.3390/cimb47050304

**Published:** 2025-04-26

**Authors:** Zahid Khan, Haroon, Yu-Feng Meng, Lian-Xi Xing

**Affiliations:** 1 College of Life Sciences, Northwest University, Xi’an 710069, China; 2 Zoology Department, University of Swabi, Anbar 23560, Khyber Pakhtunkhwa, Pakistan; 3 College of Forestry and Landscape Architecture, South China Agricultural University, Guangzhou 510642, China; 4 Shaanxi Key Laboratory for Animal Conservation, Northwest University, Xi’an 710069, China; 5 Key Laboratory of Resource Biology and Biotechnology in Western China, Northwest University, Ministry of Education, Xi’an 710069, China

**Keywords:** *Reticulitermes flaviceps*, microsatellite loci, genetic diversity, breeding system

## Abstract

The genetic diversity of 22 colonies of the termite *Reticulitermes flaviceps* was analyzed in Shaanxi and Sichuan provinces. It was found that the genetic diversity in both regions was quite similar. However, the distribution of genetic variations within the colonies was uneven. The termite colonies showed moderately high genetic diversity, a positive sign for adaptability and survival. The study also revealed a favorable mix of different genetic types within the colonies, indicating a healthy level of genetic variation. However, there was limited genetic exchange among different colonies, leading to noticeable genetic differences. When looking at the genetic structures, the colonies in Shaanxi were quite similar; those in Sichuan showed more variation, and some Sichuan colonies had identical genetic structures to those in Shaanxi. Regarding breeding systems, the colonies in Shaanxi were mainly extended families, meaning they had multiple generations living together. In contrast, most colonies in Sichuan were simple families consisting of just one generation; this difference might be due to the natural, less disturbed environments in Shaanxi, which support more extensive and complex colonies. On the other hand, the urban environments in Sichuan, with their intricate cement structures, made it difficult for termite colonies to expand. Overall, the study highlights the genetic diversity and breeding strategies of *R. flaviceps* in different environments, providing insights into their adaptability and survival mechanisms.

## 1. Introduction

Biological infestations frequently result in significant economic and environmental challenges. Social insects, particularly termites, can spread to new areas through dispersion flight and human activities, posing severe threats to urban structures [1,2,3]. The success of these infestations is attributed mainly to the complex breeding systems of termites [4]. Factors such as the number of reproductives within a colony, nest establishment patterns, mating systems, and dispersal flight influence new habitats [4,5]. These breeding systems can adapt to new environmental and ecological conditions, enhancing the invasive potential of termites [5]. Therefore, a comprehensive study of the breeding systems of subterranean termite, *R. speratus*, is important for understanding the relationship between social structure, dispersal mechanisms, and infestation success [6].

*Reticulitermes* is among the most destructive subterranean termite genera affecting buildings and forests [7]. All *Reticulitermes* species initially find their colonies through a pair of dealates, known as the primary reproductives [8]. Over time, secondary reproductives, or neotenics, which develop from nymphs or workers, replace the primary reproductives [8,9]. Neotenics are flightless and have limited dispersal capabilities, which can lead to inbreeding within the colony as they remain for mating purposes [10]. Primary reproductives lead colonial expansion and form interconnected breeding centers, resulting in extended families [4,11]. Various tasks, including worker reproduction and feeding behaviors, influence the complex colonial structure of termites [12]. If three or more adults start a colony or a pair of alates comes in and founds a colony with others, the reproductive strategy may result in a mixed family [8,13]. The cryptic nature of termites has importantly restricted our basic knowledge of termite ecology [14]. Many *R. flavipes* individuals live in logs and protect their foraging tunnels, making it challenging to study their social and spatial structures [15].

*Reticulitermes* termites are becoming popular as model organisms for investigating how genetic variation is affected by reproduction and dispersal [16]. Advances in molecular techniques now enable researchers to use genetic markers to examine the population structure of termites [5]. Recently, extensive research has been conducted on the population genetics of subterranean termites [17]. Small-scale genetic studies have focused on describing breeding systems and delineating foraging areas of *R. flavipes* and *R. hageni* in both forested and urban environments [18]. Polymorphic microsatellite markers have been widely used to investigate genetic variation within and among populations of termites [18,19,20]. Significant variations have been observed in the breeding level and structure of colonies in the subterranean termites *R. grassei* and *R. flavipes* [21]. These variations include different proportions of pedigree structures, such as the number of reproductive individuals in a nest and their kinship levels [4]. Enzyme and mtDNA markers have been used to study the breeding systems of two *R. flavipes* Kollar [22] populations in Massachusetts, where wet soil limits termite foraging. The colonies consisted of approximately 60% extended families and 40% simple families [23]. In contrast, another site with porous soil had colonies consisting of 50% extended families and 50% mixed families [19,23]. In another study on *R. flavipes* in central North Carolina, 75% of the 126 colonies were simple families, while 24% were extended families established by secondary reproductives [19]. These findings highlight the diversity of breeding systems and the influence of environmental factors on colony structure and genetic variation [15].

A diverse breeding system increases subterranean termite infestations in new ecosystems, helps to start colonies, and distributes these colonies through alate production [5]. Moreover, human activities might facilitate the transportation of termites, resulting in the establishment of large colonies in new regions [24]. The reproductive structure and dispersal patterns of colonies, whether through natural flight, underground budding nearby, or human mediation, contribute to the establishment of complex termite populations [25]. These factors influence inbreeding levels and genetic differentiation among populations [26]. Microsatellite markers are highly polymorphic, broadly distributed, and co-dominant, and follow Mendelian inheritance. They are widely used to evaluate population structure, construct genetic maps, and study the genetic diversity of termites [27,28]. Microsatellite markers have played important roles in past termite studies, such as inferring termite foraging behaviors and studying the nest structure of *R. flavipes* in North America [29]. Further migration and breeding system plasticity of the termite have been tested by five pairs of microsatellite primers [30]. The findings revealed that colony populations displayed increased migration rates in response to disturbances, which impacted colony size, individual counts, and breeding structures [30]. These studies highlight the importance of the reproductive and dispersal mechanisms of termites in attempts to understand their population dynamics and infestation potential.

*Reticulitermes flaviceps* Oshima [31] (Blattodea: Rhinotermitidae) is widely distributed in the temperate and subtropical regions of China [12]. This subterranean termite prefers humid environments and exhibits resistance to low temperatures as it primarily colonizes wood or soil, posing serious threats to buildings [8,32,33]. Genetic diversity is a major indicator of termite evolution and environmental adaptation [4,34,35]. In termites, the distribution of genetic diversity at the population level is often linked to the genetic structure at the colony level [5,36]. Therefore, analyzing the genetic structure at the colony level is essential [26]. The social structure and breeding systems of *R. flaviceps* serve as a model for potential pests. Based on previous research, in our study, microsatellite markers are used to investigate the genetic diversity, breeding systems, and gene flow among different colonies of *R. flaviceps* in Shaanxi and Sichuan provinces. To assess the negative impacts of breeding and colony dynamics, laboratory and field investigations are required. This study aims to examine the genetic diversity, structure, and breeding strategies of *R. flaviceps* populations in natural and urban environments across two provinces. By assessing colony distribution, gene flow, and adaptive traits, we aim to unveil the genetic mechanisms underlying colony formation and expansion. The findings may contribute to the development of effective strategies for controlling and mitigating the impact of this termite pest on urban ecosystems.

## 2. Materials and Methods

### 2.1. Sampling Sites

Shaanxi Province features mountainous regions and fertile plains famous for agriculture and forests that are less disturbed by urban development. It has warm summers and cold winters, providing a favorable environment for maintaining complex colony structures in natural habitats. Shaanxi’s annual average temperature is 14.5 °C (−4.4 °C min to 32.8 °C max) and average humidity is 68% (least humid ~61% and most humid ~77%). Sichuan Province is characterized by mountains and plateaus and is known for dense urbanization and industrial development. The climate is more humid and subtropical, with heavy rainfall, especially during the summer months. Sichuan’s annual average temperature is 16.5 °C (3 °C min to 30 °C max) and annual average humidity is 83% (least humid ~77% and most humid ~86%).

### 2.2. Termite Collection

We collected 22 colonies of *R. flaviceps* in rotten logs in natural environments or urban areas from Shaanxi Province and Sichuan Province. There were 7 colonies in Shaanxi numbered 1–7, and 15 colonies in Sichuan numbered 8–22. Both the soldiers and the workers were immersed in 100% ethanol and stored at −20 °C (Table 1). The workers were used for DNA extraction.

### 2.3. DNA Extraction

The experiment was conducted with 15 workers from each of the colonies; the abdomens were removed from workers to prevent the impact of the intestinal microflora. The TIANamp Genomic DNA Kit (Tiangen; Beijing; China) was used to extract genomic DNA from the homogenized cells (all somatic cells, such as neural, muscular, epidermal, and hemocyte cells) of the head and thorax, which was subsequently visualized using agarose gel electrophoresis [37]. We stored the DNA at a temperature of −20 °C under normal conditions.

### 2.4. SSR Primers and PCR Amplification Reactions

In this experiment, six pairs of primers were selected: Rs03, Ra70, Ra116, Rs76, Ra79, and Ra50 (Table 2) [38,39,40]. The optimal primer annealing temperature for PCR amplification of *R. flaviceps* DNA was found utilizing a 10 μL reaction mixture comprising 5 μL of 2× Taq PCR Master Mix, 3.4 μL of ddH_2_O, 0.4 μL of each negative and positive primer, and 0.8 μL of template DNA [37,41]. The reaction protocol began with a pre-denaturation step at 95 °C for 5 min to completely denature the DNA template. Subsequently, 30 amplification cycles were conducted, each including a 30 s denaturation at 95 °C, a 30 s annealing step at the specified temperature for primer binding, and a 30 s extension at 72 °C. Following 30 cycles, a final extension was conducted at 72 °C for 10 min.

Six pairs of primers were amplified for all individuals, and the amplified products were sent to Shanghai Biotech for capillary electrophoresis. The resulting data were processed using the software GeneMarker v2.2.0 (SoftGenetics@, State College, PA, USA) to obtain the fragment sizes of each individual as raw data. Hardy-Weinberg equilibrium calculations were performed on data from different loci between nests using the software Fstat 2.9.3.2 (Lausanne, Switzerland) [42]. Moreover, GenALEx 6.5 software (Peakall & Smouse, Australian National University, Canberra, Australia) was used to calculate the genetic diversity index for each locus, including allele number (Na), effective allele (Ne), Shannon information index (I), observed heterozygosity (Ho), expected heterozygosity (He), and gene flow parameters (Nm) [5,40,43,44]. The polymorphic information content (PIC) of each locus was calculated using Cervus V3.0 software (Field Genetics Ltd., London, UK) [45]. The genetic structure among different nests was analyzed using the software Structure 2.2 (Pritchard, Stephens, and Donnelly, the University of Chicago, Chicago, IL, USA), and the level of gene flow (Nm) between nests was assessed. We followed the method of *F*-statistical classification proposed by Deheer and Vargo [15] to analyze colony structure and breeding systems. The Fstat 2.9.3.2 software (Goudet, University of Lausanne, Switzerland) was also used for genetic structure analyses. The *F*-statistics, with symbols I, C, and T, displayed genetic variation among individuals, colonies, and the total population [11]. We obtained a 95% confidence interval by bootstrapping 10,000 times at the locus and testing significance among individuals with allele replacement. *F*_IC_, the inbreeding coefficient at the colony level, indicated the spatial distribution and number of reproductives, plus mating patterns. As the reproductive number increased, *F*_IC_ showed significant negative values in simple nests, approaching zero. Positive *F*_IC_ values showed intra-colony mating among reproductives or inter-colony mixing. *F*_IT_*,* similar to *F*_IS_*,* measured deviations from random mating in a population. The T was the same as S, and there was no subgroup structure in the analysis [46]. The *F*_ST_ value of *F*_CT_ and genetic differentiation among colonies were comparable. The affinity coefficient (r) of individuals among colonies was calculated [47].

## 3. Results

### 3.1. Outcome of Gel Electrophoresis

Agarose gel electrophoresis of termite DNA samples revealed distinct, clear bands with no trailing, indicating high-quality, intact DNA and effective extraction (Figure S1). Polyacrylamide gel electrophoresis confirmed successful PCR amplification and primer polymorphism, showing genetic variation and demonstrating that the DNA is suitable for future genetic research (Figure S2).

In partnership with Shanghai Biotech (Shanghai Biotechnology Corporation, Shanghai, China), the ABI3100 DNA Analyzer analyzed PCR-amplified termite DNA samples quickly and sensitively using capillary electrophoresis (CE). CE with great resolution improved the genetic analysis by distinguishing DNA fragments, even by a single base pair. This automated, high-throughput method provided reliable, consistent data for genotyping using minimum DNA.

### 3.2. Genetic Diversity

The genetic diversity of *R. flaviceps* in Shaanxi and Sichuan Provinces indicated significant variations between the two populations using GenALEx 6.5 software. A total of 105 individuals from the seven colonies in Shaanxi province were analyzed to obtain the data. The number of alleles (Na) ranged from 2.429 to 3.000; the average number of alleles was 2.738 ± 0.132. The number of effective alleles (Ne) was in the range of 1.832–2.293, and the average effective allele was 2.057 ± 0.094. Both were lower than the number of alleles, indicating that the number of alleles at the six loci was found in these seven colonies. The distribution was considered uneven. The observed heterozygosity (Ho) ranged from 0.390 to 0.610, with an average of 0.494 ± 0.037. The expected heterozygosity (He) ranged from 0.420 to 0.537, with an average of 0.472 ± 0.024, which was lower than the observed heterozygosity. This indicates that heterozygotes were predominant among the seven populations. Shannon’s information index (I) ranged from 0.690 to 0.890, and the average was 0.773 ± 0.044 (Table 3).

Among colonial populations in Sichuan (Na), the total number of alleles ranged from 1.933 to 3.600, with an average of 2.889 ± 0.130. The number of effective alleles (Ne) was 1.589–2.544, and the average value was 2.107 ± 0.095. Similar to the situation in Shaanxi, the values were lower than the number of alleles (Na), and the alleles at six loci were unevenly distributed across the 15 colonies. The observed heterozygosity (Ho) ranged from 0.329 to 0.622, with an average of 0.512 ± 0.038. The expected heterozygosity (He) was also greater than 0.3, and it ranged from 0.286 to 0.514. The average expected heterozygosity was 0.436 ± 0.025, which was also lower than the observed heterozygosity (Ho). Shannon’s information index (I) was up to 0.938, the lowest was 0.448, and the average was 0.752 ± 0.046. It showed that the 15 colonies in Sichuan, like the nest group in Shaanxi, have rich genetic diversity and were at a medium level. The minimum polymorphism information content (PIC) was 0.627, the maximum was 0.897, and the average was 0.777 ± 0.039, all of which were greater than 0.5. As a result, these six loci in the Sichuan colonies were also highly polymorphic (Table 3). The results revealed that the Sichuan population exhibits a somewhat higher level of allelic diversity, indicating a greater degree of genetic variety in comparison to the Shaanxi population. These varieties were essential for the flexibility and adaptability of termite populations in their specific habitats.

Six microsatellite loci were used to investigate the *F*-statistics and gene flow (Nm) in *R. flaviceps*, revealing different levels of genetic divergence and inbreeding within the population. The inbreeding coefficient within individuals (*F*_IS_) varied from −0.275 to 0.131, with an average of −0.133 ± 0.058, indicating a general surplus of heterozygotes. The population showed moderate inbreeding at the population level, as indicated by the overall inbreeding coefficient (*F*_IT_) ranging from 0.286 to 0.536, with a mean of 0.376 ± 0.036. The genetic variation among populations, as measured by *F*_ST_, varied from 0.405 to 0.483, with an average of 0.450 ± 0.014, indicating significant genetic divergence. The estimates for gene flow (Nm) ranged from 0.267 to 0.368, with an average of 0.309 ± 0.018, indicating that there was only a small amount of gene flow occurring among groups (Table 4). The findings of this study revealed substantial genetic differentiation and a moderate level of inbreeding among the populations of *R. flaviceps* that were examined.

### 3.3. Hardy Weinberg Test

The Hardy-Weinberg equilibrium test results for six microsatellite loci in 22 nests of *R. flaviceps* revealed varying degrees of genetic equilibrium across different loci and colonies, as determined using Fstat 2.9.3.2 software (Table 5). Notably, several loci, such as Ra116, Ra070, and Rs03, showed significant deviations from Hardy-Weinberg equilibrium in multiple colonies, indicated by *p*-values less than 0.05 (e.g., Ra116 in colonies 2, 6, 8, 12, and 13 from Sichuan, and 17 from Shaanxi; Ra070 in colonies 3, and 8 from Sichuan, and 22 from Shaanxi; and Rs03 in colonies 10 and 12 from Sichuan, and 22 from Shaanxi). These deviations suggested potential factors such as non-random mating, genetic drift, or selection pressures affecting these loci. Additionally, some loci were monomorphic in certain colonies, indicated by the “-” symbol, implying no genetic variation at those positions within those colonies. Overall, the data highlighted the genetic diversity and potential evolutionary dynamics within the termite populations studied.

### 3.4. Genetic Structure of R. flaviceps

**Figure 1:**
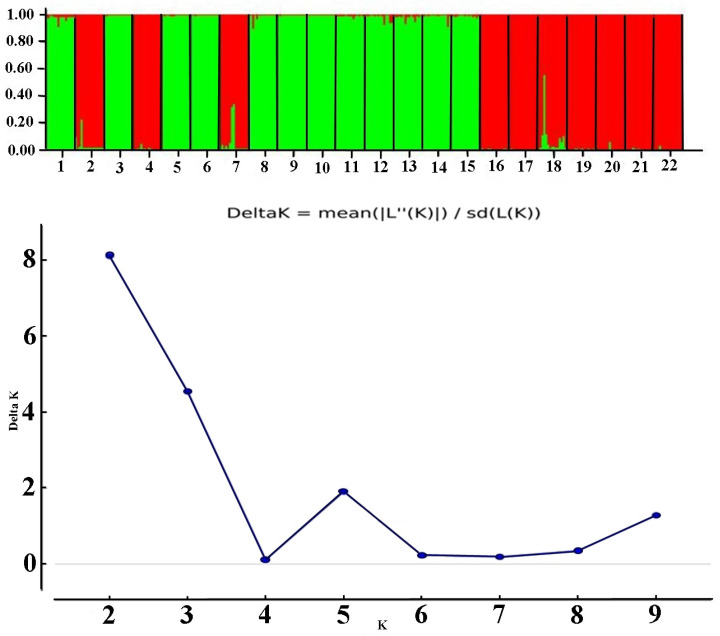
Population genetic structure analysis of *R. flaviceps* sampled from different geographical regions based on microsatellite loci. Colonies 1 to 15 were from Sichuan Province, and Colonies 16 to 22 were from Shaanxi Province. Each vertical bar shows a colony’s genetic makeup, with green and red indicating the proportion of two genetic clusters.

The genetic structural analysis of *R. flaviceps* using the software Structure 2.2 revealed that using the Bayer clustering method, the most appropriate number of genetic clusters (K) was 2. This conclusion was based on the ΔK value reaching its maximum at K = 2. The bar graph in Figure 1 illustrated the genetic composition of termite colonies from different geographical regions, with colonies 1–15 representing Sichuan Province and colonies 16–22 representing Shaanxi Province. Each vertical bar displayed the genetic composition of a termite colony, with varying proportions of green and red, indicating the presence and proportion of two different genetic clusters within each colony. The predominance of one color over another in certain bars suggested that some colonies were more genetically similar within one cluster than mixed between two. This visual representation helped to understand the genetic differentiation and structure of *R. flaviceps* populations across different regions, providing insights into their genetic diversity and evolutionary dynamics.

### 3.5. Colony Structure and Breeding System of R. flaviceps

The colony structure and breeding system analysis of *R. flaviceps* revealed distinct differences among Shaanxi and Sichuan Provinces populations. In Shaanxi, all seven colonies (100%) were classified as extended families, indicating complex genetic structures inconsistent with simple family genotypes. In contrast, the Sichuan population predominantly consisted of simple families, with 12 out of 15 colonies (80%) fitting the simple family model, where worker genotypes aligned with those expected from primary reproductives. Only 3 colonies (20%) in Sichuan were extended families, and no mixed families were observed in either population (Table 6 and Table 7). These findings suggested significant regional variation in the breeding systems and colony structures of *R. flaviceps*, potentially influenced by local environmental factors or genetic diversity. When five or more alleles were observed at one or more loci, the colonies were classified as mixed families. In these mixed families, there were multiple primary reproductives and a large number of secondary reproductives. On the other hand, in extended families, there was only one pair of primary reproductives, along with the secondary reproductives they produced.

Table 7 showed F-statistics, relatedness coefficients for *R. flaviceps*, and a simulation of possible termite breeding systems. In this context, X represented the number of generations of second-generation reproductives, Nf denoted the number of female supplementary reproductives per generation, Nm represented the number of male supplementary reproductives per generation, and *p* indicated the proportion of workers from different colonies after fusion. The inbreeding coefficient within colonies (*F*_IC_) was equivalent to *F*_IS_ and was crucial for analyzing the reproductive structure of the colony. The Table 7 showed that the *F*_IC_ for simple families was −0.215, indicating a significant excess of heterozygotes. In extended families, the *F*_IC_ was also negative, approaching zero as the number of reproductives increased. Additionally, simple families exhibited the highest genetic differentiation coefficient (*F*_CT_) and the highest relatedness coefficient among workers in the colony.

## 4. Discussion

The breeding systems of *R. flaviceps* in urban areas of Shaanxi and Sichuan may contribute to serious economic impacts, biodiversity loss, and ecosystem degradation due to biological infestations, as previous study has reported [36]. The average number of alleles per locus (Na) was 2.738, with an effective number of alleles (Ne) of 2.057 and a Shannon’s Information Index (I) of 0.773 in Shaanxi. The measured level of genetic diversity was 0.494, as indicated by the observed heterozygosity (Ho). The average Polymorphic Information Content (PIC) was 0.638, and the expected heterozygosity (He) was 0.472, indicating that among the seven colony populations, heterozygotes were mainly dominant. In addition, when the heterozygosity is expected to be greater than 0.3, the genetic diversity of the population can be considered to be good [48]. Therefore, the genetic diversity of seven termite colonies in Shaanxi was better. Among the 15 colony populations in Sichuan, greater genetic variety was found, characterized by an average Na of 2.889, Ne of 2.107, and I of 0.752. The Ho value was 0.512, the He value was 0.436, and the average *P*_IC_ value was 0.777. Similarly, Zhao et al. [40] reported a related study on *R. aculabialis*, noting that the genetic diversity of populations in Xi’an and Nanjing was similar, with moderate but relatively low levels of variation—consistent with the current findings. There were few related studies conducted in the past [49,50,51,52].

In our experimental work, the *F*_IS_ value of the inbreeding coefficient was −0.133, indicating that the heterozygote was abundant in the termite nest group, which was consistent with the expected heterozygosity in all colonies in the two places. The inbreeding coefficient (*F*_IT_) of the total population was 0.376 in the termite colony. However, the genetic differentiation coefficient (*F*_ST_) ranged from 0.405 to 0.483, and the average value was 0.450 in this experiment, which was greater than 0.15. The gene flow index (Nm) was 0.309, the range was between 0.267 and 0.368, and the value was less than 1. The data in the results were obtained by analyzing the total of 330 individual *F* statistics and gene flow (Nm) for 22 colonies in two places. The inbreeding coefficient *F*_IS_ (−1 to 1) indicates heterozygosity in a colony due to non-random mating. A positive *F*_IS_ (>0) reflects heterozygote deficiency, while a negative *F*_IS_ (<0) suggests out-crossing and preserved heterozygosity [20]. *F*_ST_ measured population variations from 0 (random mating) to 1 (total isolation). Values greater than 0.15 indicated considerable variation, whereas 0.05 to 0.15 suggested moderate variation. When the population was at a lower level of differentiation, the index will be less than 0.05 [53,54]. Nm measures the flow of genes between populations, thereby showing the migration-mediated gene exchange. Nm can also reflect the differences between populations.

In contrast, the breeding system in *Labiotermes labralis* colonies was also found in Rocoucoua, Patagai, and Cacao, French Guiana. The *F*_IC_ ranged from −0.036 to −0.237, the *F*_IT_ ranged from 0.112 to 0.424, and the *F*_CT_ ranged from 0.213 to 0.445, and there are similar studies to the current findings [5,15,49,55,56,57]. This study indicated that *R. flaviceps* may exhibit greater genetic variation among colonies, possibly because of environmental stresses or limited gene flow.

As can be seen, only 16 (12.1%) of the 132 tests deviated from the Hardy-Weinberg equilibrium (*p* < 0.05). Most of them showed a phenomenon consistent with the Harvard equilibrium, and no one site always demonstrated a deviation from the Harvard balance. Therefore, all colonies belong to natural mating, and these six loci data were used for genetic diversity analysis. The basis of was the random mating of natural populations. This inheritance will continue from one generation to the next [58].

Alates, primary and secondary reproductives, or complementary reproductives with the same genotype established simple families. In extended families, workers had different genotypes and showed progeny from at least three reproductives; there were no more than four alleles at any locus, suggesting all secondary reproductives were offspring of an alate pair. Mixed families were identified by five or more alleles at any microsatellite locus, displaying unrelated offspring from same-sex reproduction. There were two or more unrelated offspring reported from same-sex reproduction [15,20].

In this study, the seven colonies from Shaanxi belonged to the extended family. Of the 15 colonies in Sichuan, 12 were simple colonies, whereas only 3 were extended colonies. Perhaps this is because all the extended colonies were located in their natural habitat. The habitats were suitable for termite survival, allowing the colonies to expand. The 12 simple families in Sichuan were collected from the urban areas. The majority of urban structures were made of rigid cement concrete, which was not conducive to the expansion of termite colonies, and more related to the previous study of Dronnet et al. [5]. Similarly, in North Carolina, 75% of *R. flavipes* colonies were simple families, 25% were extended, and 1–2% were mixed [15]. However, the low frequency of mixed families in these studies indicated that mixing workers from two different colonies was not a common occurrence in *R. flaviceps*.

Termites established mixed colonies due to mixing between workers from nearby colonies, which could have increased with foraging range expansion, especially the lack of nestmate discrimination in *R. flavipes* [56]. However, foragers of *Odontotermes formosanus* from nearby colonies of the same species or with other species have high genetic variation and, therefore, appear to maintain separate foraging areas [59]. While termite, *C. formosanus,* from different colonies had no agonistic behaviors, as no aggression was observed in the same colony or even between neighbor colonies of the same species, except the presence of a distinct colony odor, which indicated that colony integration was possible [60]. Which may also happen in *R. flaviceps* colonies.

DeHeer and Vargo [29] investigated the fusion of 8 colonies of *R. flavipes*, and in all cases, the fused colonies had the same or nearly identical mitochondrial DNA haplotype. Multiple pairs of *Macrotermes michaelseni* can coexist in a single colony, and the success rate of colony establishment by 4–5 pairs of dealates was significantly higher than that by 1–2 pairs. Therefore, the formation of polygynous colonies may also be due to the co-establishment of multiple alate pairs when they are flying [61].

Vargo et al. [21] analyzed the effects of biological variables, latitude, and climatic conditions (cold and moist habitats) on the colony breeding systems of the termite, *R. grassei* (France), and *R. flavipes* populations (USA). Similarly, these biological variables can also affect the colony breeding systems of the termite, *R. flaviceps*, in Xi’an and Sichuan. The number of polygynies (different queens) in *M. michaelseni* termite colonies varies depending on geography [61]. The genetic diversity of *R. flaviceps* provided knowledge about the breeding systems of social pests, which were constantly changing. We studied the genetic structure of *R. flaviceps* in natural and urban environments, along with its reproduction rate and the factors that facilitate its distribution to new areas. These findings are consistent with those of a previous study conducted by Dronnet et al. [5].

## 5. Conclusions

This study examined the genetic diversity and breeding systems of *R. flaviceps* across 22 colonies in various environmental situations in Shaanxi and Sichuan Provinces. The analysis indicated that the majority of colonies in Shaanxi were composed of extended families, which contributed to greater genetic diversity, as evidenced by the observed heterozygosity and allele frequencies. In particular, Shaanxi termite colonies showed a more complex family structure that survived over several generations, enhancing adaptation. On the other hand, Sichuan’s colonies were more simple family structures, likely brought about by environmental stresses and urbanization; this factor potentially restricted their genetic diversity and potential for expansion. The limited ability of *R. flavipeps* to spread and their dependence on human activity for transportation were highlighted by the genetic differentiation coefficients, which showed a notable absence of gene flow among the colonies. The Sichuan population has more allelic diversity than the Shaanxi population, according to an analysis of several genetic factors. This variation is important for a colony’s ability to survive in its environment. This study increases our knowledge of how ecology and genetics interact in termite populations, providing important methods of control for pest species that seriously damage urban ecosystems and infrastructure.

## Figures and Tables

**Table 1:** Sample information and collection site of *R. flaviceps.*

Colony	Provinces	Collection Location	Sampling Time	Coordinates	Type of Habitat
1	Shaanxi	Ziyang Wenbi Mountain	2013.11.24	108.32′45″, 32.31′5″	Natural habitat
2		Ziyang Donghe	2013.11.24	108.17′20″, 32.28′31″	Urban areas
3		Ziyang county town	2013.11.24	108.32′33″, 32.31′30″	Urban area
4		Zhashui county town	2013.11.24	109.7′9″, 33.41′27″	Natural habitat
5		Zhoutaizhuang	2016.09.09	109.2′19″, 32.43′9″	Natural habitat
6		Chaojiazhuang	2016.09.09	107.58′51″, 33.26′21″	Natural habitat
7		Xunyang county Xiangubian	2015.12.21	109.9′, 33.7′42″	Natural habitat
8	Sichuan	Dayi	2014.11.10	107.58′51″, 33.26′21″	Natural habitat
9		Wenjiang	2015.12.10	103.51′48″, 30.41′17″	Urban area
10		Pengzhou	2014.11.10	103.57′2″, 31.0′6″	Urban area
11		Dujiang Dam	2014.11.11	103.37′3″, 31.0′28″	Natural habitat
12		Xinjin	2015.11.23	103.49′2″, 30.24′52″	Urban area
13		Pujiang county	2013.10.25	103.30′4″, 30.11′4″	Natural habitat
14		Qingcheng Mountain	2016.10.15	103.34′30″, 30.54′4″	Natural habitat
15		Jinjiang District	2016.11.14	104.5′23″, 30.39′43″	Urban area
16		Ancestral Temple of Zhaogong	2017.11.15	103.33′5″, 30.57′33″	Natural habitat
17		Huangnigou	2017.11.15	103.33′2″, 30.57′19″	Natural habitat
18		Tumenzi	2017.11.15	103.54′22″, 29.32′49″	Urban areas
19		Pingshiban	2017.11.16	103.35′14″, 30.59′50″	Natural habitat
20		Longfeng Rock	2017.11.14	103.32′59″, 30.57′57″	Natural habitat
21		Zhaogong Mountain 1	2017.11.14	103.33′17″, 30.57′40″	Natural habitat
22		Zhaogong Mountain 2	2017.11.14	103.33′17″, 30.57′40″	Natural habitat

**Table 2:** Information on microsatellite loci and annealing temperature.

Primer	Primer Sequence	Core Repeat Unit	Annealing Temperature	Fluorescent Group
Ra79	F: TACCCTGTGGAGAACTCGCT	(GAAT) 9	55.6 °C	HEX
	R: AATGACCTTCTTGGGCGTTT			
Rs76	F: AATCCGGGGAATTTCTTGAC	(AGTT) 8	55.6 °C	ROX
	R: CTGCATAACGATGTCTGCGT			
Ra50	F: TCCAGTTGTCACTTCGACAGA	(ATGT) 15	50.3 °C	FAM
	R: GTCAAGGTCCCGTCCTGTTA			
Ra116	F: TCGACCGACTCAGTAGCCTT	(TCT )11 +(CCT)6	56.9 °C	HEX
	R: AAAGATGGAGGGACGAGGTT			
Ra70	F: TACAGAGCTTTCATGGCACG	(CTA) 12	58.2 °C	ROX
	R: AAACCTCGAAATGAGGAGGC			
Rs03	F: TCCTGACTGTACAAAGAAAAGTGG	(CT)9	55.6 °C	FAM
	R: TGGCATCAAGCTACGTATTCA			

**Table 3:** Genetic diversity index of *R. flaviceps* from two different ecological environments.

Shaanxi Province							
Locus	N	Na	Ne	I	Ho	He	PIC
Ra079	15.000	2.571	1.996	0.690	0.390	0.420	0.646
Rs076	15.000	2.857	1.832	0.728	0.524	0.429	0.460
Ra050	15.000	3.000	2.029	0.790	0.486	0.467	0.703
Ra116	15.000	3.000	2.293	0.890	0.495	0.537	0.812
Ra070	15.000	2.429	2.138	0.789	0.457	0.510	0.544
Rs03	15.000	2.571	2.051	0.753	0.610	0.471	0.662
mean	15.000	2.738	2.057	0.773	0.494	0.472	0.638
SE	0.000	0.132	0.094	0.044	0.037	0.024	0.050
**Sichuan Province**							
Ra079	15.000	3.600	2.544	0.938	0.622	0.508	0.800
Rs076	15.000	1.933	1.589	0.448	0.347	0.286	0.627
Ra050	15.000	2.667	1.966	0.729	0.622	0.448	0.816
Ra116	15.000	2.733	1.906	0.682	0.329	0.394	0.708
Ra070	15.000	3.133	2.293	0.828	0.556	0.464	0.897
Rs03	15.000	3.267	2.345	0.885	0.596	0.514	0.818
mean	15.000	2.889	2.107	0.752	0.512	0.436	0.777
SE	0.000	0.130	0.095	0.046	0.038	0.025	0.039

N—individuals; Na—average number of alleles per locus; Ne—effective number of alleles; I—Shannon’s information Index; Ho—observed heterozygosity; He—predicted heterozygosity; PIC—polymorphic information content.

**Table 4:** *F*-statistics and Nm estimates of *R. flaviceps.*

Locus	*F*_IS_	*F*_IT_	*F*_ST_	Nm
Ra079	−0.143	0.321	0.406	0.366
Rs076	−0.216	0.357	0.472	0.280
Ra050	−0.275	0.341	0.483	0.267
Ra116	0.131	0.536	0.466	0.287
Ra070	−0.095	0.415	0.466	0.286
Rs03	−0.199	0.286	0.405	0.368
Mean	−0.133	0.376	0.450	0.309
SE	0.058	0.036	0.014	0.018

F_IS_—inbreeding coefficient within individuals; F_IT_—denotes the inbreeding coefficient of the total population; F_ST_—denotes genetic differentiation coefficient; Nm—means gene flow index.

**Table 5:** *p* values of Hardy-Weinberg equilibrium for 6 microsatellite loci in *R. flaviceps.*

Provinces	Colony	Ra079	Rs076	Rs050	Ra116	Ra070	Rs03
Sichuan	1	0.054	-	0.829	1.000	1.000	1.000
	2	0.948	-	1.000	0.036 *	-	1.000
	3	-	-	0.371	1.000	0.008 *	0.151
	4	1.000	0.193	1.000	-	1.000	1.000
	5	-	0.103	1.000	0.979	0.996	1.000
	6	1.000	1.000	1.000	0.001 *	0.103	1.000
	7	1.000	-	1.000	0.721	-	0.735
	8	0.000 *	1.000	1.000	0.000 *	0.999	1.000
	9	1.000	0.989	1.000	-	0.776	0.561
	10	0.977	1.000	1.000	1.000	0.842	0.000 *
	11	0.715	-	1.000	0.721	0.824	0.090
	12	0.550	0.881	0.513	0.004 *	0.468	0.000 *
	13	1.000	0.401	0.898	0.026 *	1.000	1.000
	14	0.303	1.000	1.000	0.101	0.522	0.879
	15	0.342	1.000	0.924	0.785	0.835	0.959
Shaanxi	16	1.000	1.000	1.000	1.000	0.461	0.245
	17	0.245	0.949	0.899	0.000 *	0.881	0.989
	18	-	0.979	0.995	0.007 *	0.371	0.988
	19	0.001 *	1.000	1.000	1.000	1.000	1.000
	20	1.000	0.821	0.801	0.949	0.107	1.000
	21	0.898	1.000	1.000	0.858	0.881	0.982
	22	0.034 *	0.069	0.000 *	0.000*	0.000 *	1.000

Note: * indicates deviation from Hardy Weinberg equilibrium; - indicates that *R. flaviceps* is monomorphic at this position.

**Table 6:** Numbers of simple family and extended family of *R. flaviceps.*

Population	Colonies No.	Simple Family	Extend Family	Mixed Family
Shaanxi	7	0 (0%)	7 (100%)	0 (0%)
Sichuan	15	12 (80%)	3 (20%)	0 (0%)

**Table 7:** F-statistics and relatedness coefficients for worker nestmates of *R. flaviceps.*

	*F*_IT_	*F*_CT_	*F*_IC_	r
Shaanxi				
Extended-family colonies (*n* = 5)	0.279	0.308	−0.042	0.481
(95%CI)	0.109–0.415	0.214–0.384	−0.160–0.091	0.370–0.559
Sichuan				
Simple-family colonies (*n* = 12)	0.348	0.504	−0.215	0.687
(95%CI)	0.208–0.487	0.394–0.536	−0.353 to −0.050	0.631–0.742
Extended-family colonies (*n* = 3)	0.462	0.479	−0.033	0.655
(95%CI)	0.287–0.621	0.374–0.590	−0.322–0.235	0.528–0.779
Simulated breeding system				
(A) Simple-family colonies with				
(1) Outbred reproductive pairs	0.00	0.25	−0.33	0.50
(2) Inbred reproductive pairs	0.33	0.42	−0.14	0.62
(B) Extended family colonies with inbreeding among multiple neotenics				
(1) Nf = Nm = 1, X = 1	0.26	0.65	−0.14	0.55
(2) Nf = 2, Nm = 1, X = 3	0.52	0.59	−0.17	0.78
(3) Nf = Nm = 10, X = 1	0.33	0.34	−0.01	0.51
(4) Nf = 200, Nm = 100, X = 3	0.33	0.34	0.00	0.50
(C) Mixing between unrelated colonies				
Nf = Nm = 1, X = 3, *p* = 0.8	0.57	0.43	0.25	0.55
(D) Mixing between related colonies				
Nf = Nm = 1, X = 3, *p* = 0.9	0.66	0.64	0.04	0.77

*F*_IT_—the inbreeding coefficient of the total population; *F*_CT_—the highest genetic differentiation coefficient; *F*_IC_—the inbreeding coefficient at the colony level; r—relatedness coefficients.

## Data Availability

Experimental data are available from the corresponding author on request.

## Supplementary Materials

The following supporting information can be downloaded at: https://www.mdpi.com/article/10.3390/cimb47050304/s1.
