# Supplementary material for: Genetic Diversity and Breeding System of the Pestiferous Subterranean Termite Reticulitermes flaviceps Across Shaanxi and Sichuan Provinces
Source: Curr Issues Mol Biol. 2025 Apr 26;47(5):304. doi: 10.3390/cimb47050304 (PMC12110644; doi:10.3390/cimb47050304)
Supplement: Supplementary file 1 [file cimb-47-00304-s001.zip › cimb-3546845-supplementary.pdf]

Supplementary Materials

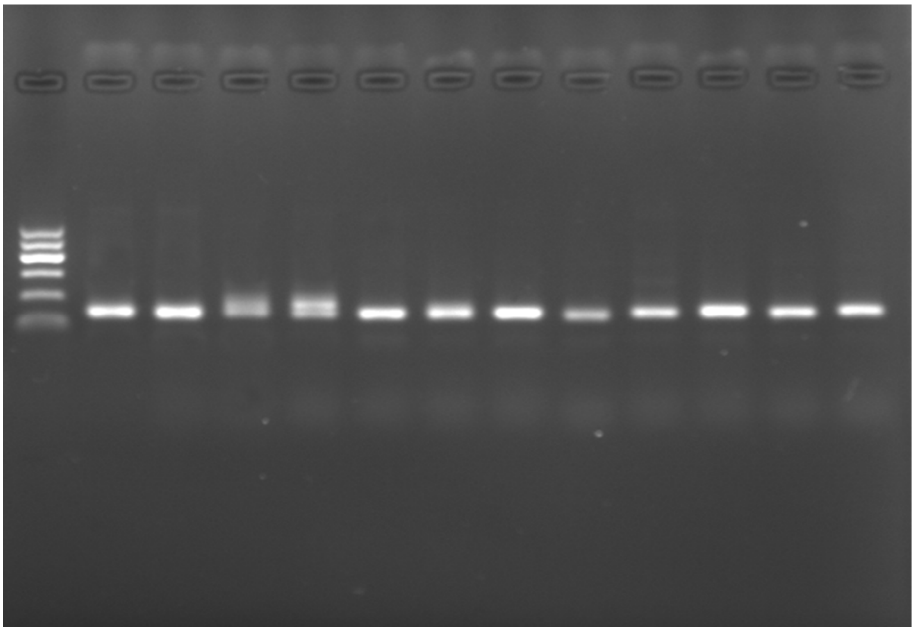

Supplementary Figure S1. Agarose gel electrophoresis of genomic DNA.

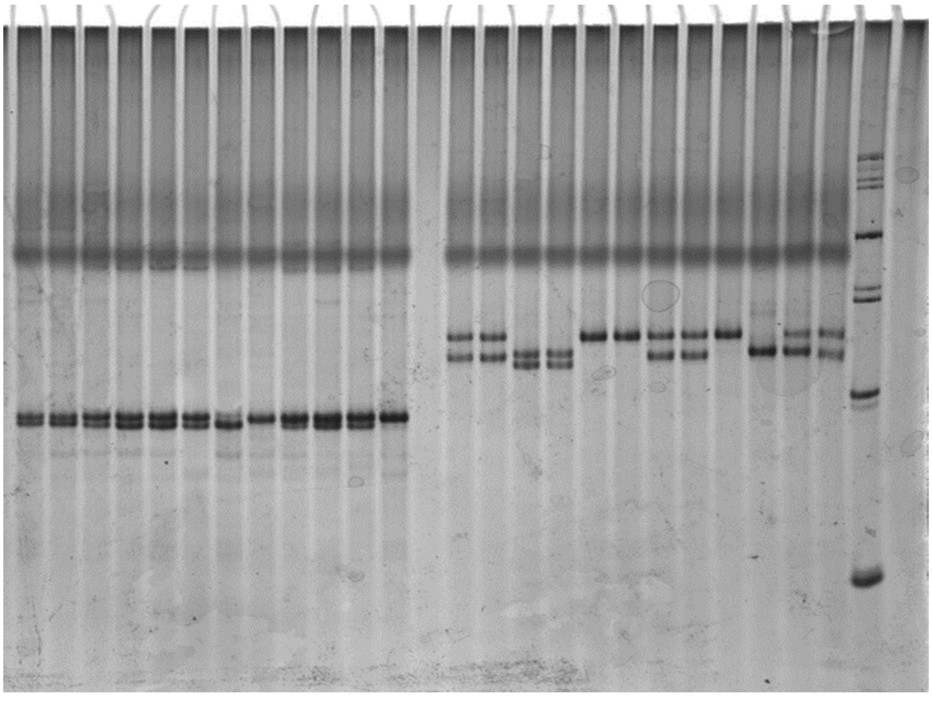

Supplementary Figure S2. Polyacrylamide gel electrophoresis in *R. flaviceps.*
